# Genetic analysis of mitochondrial DNA from ancient *Equus caballus* bones found at archaeological site of Joseon dynasty period capital area

**DOI:** 10.5713/ab.21.0500

**Published:** 2022-03-01

**Authors:** Jong Ha Hong, Chang Seok Oh, Sun Kim, In Uk Kang, Dong Hoon Shin

**Affiliations:** 1 Institute of Korean Archaeology and Ancient History, Kyung Hee University, Seoul 02447, Korea; 2 Department of Mortuary Science, College of Bio-Convergence, Eulji University, Seongnam 13135, Korea; 3 Research Institute of Buddhist Cultural Heritage, Seoul 03147, Korea; 4 Comparative Anatomy Lab, Institute of Forensic and Anthropological Science, Seoul National University College of Medicine, Seoul 03080, Korea

**Keywords:** Ancient DNA, *Equus caballus*, Horse, Joseon Period, Mitochondrial DNA, South Korea

## Abstract

**Objective:**

To understand the domestication and spread of horses in history, genetic information is essential. However, mitogenetic traits of ancient or medieval horses have yet to be comprehensively revealed, especially for East Asia. This study thus set out to reveal the maternal lineage of skeletal horse remains retrieved from a 15th century archaeological site (Gongpyeongdong) at Old Seoul City in South Korea.

**Methods:**

We extracted DNA from the femur of *Equus caballus* (SNU-A001) from Joseon period Gongpyeongdong site. Mitochondrial (mt) DNA (HRS 15128-16116) of *E. caballus* was amplified by polymerase chain reaction. Cloning and sequencing were conducted for the mtDNA amplicons. The sequencing results were analyzed by NCBI/BLAST and phylogenetic tool of MEGA7 software.

**Results:**

By means of mtDNA cytochrome b and D-loop analysis, we found that the 15th century Korean horse belonged to haplogroup Q representing those horses that have historically been raised widely in East Asia.

**Conclusion:**

The horse is unique among domesticated animals for the remarkable impact it has on human civilization in terms of transportation and trade. Utilizing the Joseon-period horse remains, we can obtain clues to reveal the genetic traits of Korean horse that existed before the introduction of Western horses.

## INTRODUCTION

Among the most pivotal events in human history was the domestication of wild animals. Once animals could be utilized as livestock, they provided daily labor as well as necessities in the form, for example, of clothing and food, all for the maintenance of human civilization [1–3]. The horse has a unique position in history, as it had perhaps the most profound impact on human society, having decisively influenced transportation, trade, and human life itself [4]. The advent of interregional communication, the expansion of trade, the enhancement of agricultural productivity, and even the spread of diseases - all were accelerated using by the use of domesticated horses [5].

The horse, despite its relatively low population density, has shown the widest geographic distribution among livestock [3]. During the Pleistocene, large herds of *Equus ferus* (wild horses) roamed the plains of Asia, Africa, Europe, and North America, though their habitats had become somewhat reduced by the end of the Ice Age [6]. The earliest domestication of the horse seems to have occurred on the Kazakhstan steppes between the fifth and fourth millennia BCE [7]. The overall genetic and archaeological evidence obtained to date shows evidence of several horse domestications in a variety of regions [4,8–15].

Over the past several decades, genetic analyses on mito chondrial DNA (mtDNA) have been performed for different horse breeds. The first-ever report of full-sequence horse mtDNA (16,660 bp) was that of Xu and Arnason [16]. From then on, many researchers have continued to conduct mtDNA research on the following subjects: Japanese domestic horse [17], Jeju Island horse [18], African ass, plains zebra and horse [19], equid remains from archaeological sites of late-Pleistocene deposits [8], Oriental and European breeds along with American mustangs [9], German heavy draught horse breeds [20], Iberian horses [21], indigenous ancient and modern Irish horse populations [10], ancient Chinese domestic horse [22], domestic horses in modern China [11], ancient Iberian horse remains [12], whole mitochondrial genomes of domestic and *E. przewalskii* horses [4], as well as mitogenomes of modern horses in Asia, Europe, and the Americas [13]. Recently, Cieslak et al [23], Achilli et al [13], and Yang et al [15] all reported that horses could be genetically classified into several major mitochondrial lineages (A to G) and 19 clusters.

Such genetic information is crucial to any comprehensive understanding of the domestication of horses and their subsequent geographical spread in history. However, those studies notwithstanding, the accumulated mitogenetic data on ancient or medieval horses remains insufficient. This is true especially for Korean horses. Our current study therefore was undertaken to uncover genetic information on skeletal horse remains collected at a 15th century archaeological site located in the capital (Old Seoul City) of the Joseon Kingdom (1392–1910 CE). We endeavored to obtain insights into the genetic traits of the medieval Korean horse that had prevailed in Korea prior to the introduction of Western horses in the 20th century.

## MATERIALS AND METHODS

### Sample collection

**Figure 1:**
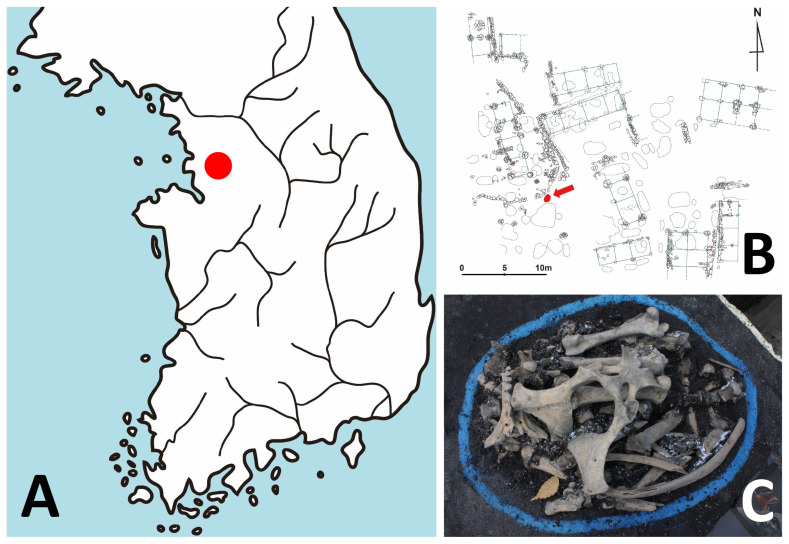
Archaeological context of the ancient horse bone in this study. (A) Geographic location of Gongpyeong-dong site (red dot) in Seoul, South Korea. (B) Map of archaeological site. The pit where horse bones were found is marked by a red arrow. (C) The horse bones used for this study.

The Gongpyeongdong archaeological site is located in the area of Old Seoul City (Hansung) of the Joseon Dynasty period (Figure 1A). The archaeological investigation was conducted by the Research Institute of Buddhist Cultural Heritage (Seoul, South Korea). Among the excavation sites including a residence, lavatory, drainage facility, and furnace, among others, animal bones were retrieved from 15th century pits (#1 to #3; layer VI) (Supplementary Table S1) [24]. A zooarchaeological examination confirmed the bones to be those of a horse [24] (Figure 1B, 1C). Among the samples, SNU-A001 (#1 of layer VI) was used for ancient DNA analysis. We followed the instructions of Hofreiter et al [25] and Ho and Gilbert [26] in protecting the authenticity of our ancient DNA (aDNA) analysis. Briefly, during the sampling and subsequent aDNA analysis, no one other than one researcher (Hong JH) doing lab work was allowed to be in contact with the samples. Also, our molecular work was carried out in a specialized facility exclusively dedicated to aDNA analysis. The tools used in the sampling and aDNA analysis, moreover, were sterilized, and the researcher wore contamination-free gloves, masks, and gowns [26].

### aDNA extraction, purification, and isolation

We used the femurs of *Equus caballus* (SNU-A001) as the specimen for our aDNA analysis. DNA extraction was performed following the protocol described in one of our earlier papers [2]. Briefly, the bone surface was abraded, irradiated under ultraviolet light, and subsequently treated in sodium hypochlorite (5.4%). After being dried, the sample was powdered with SPEX 6750 Freezer/Mill (SPEX SamplePrep, Metuchen, NJ, USA). A portion of powdered sample (0.3 g) was incubated in lysis buffer (pH 8.0). DNA was extracted and purified with phenol-chloroform-isoamyl alcohol (25:24:1), chloroform-isoamyl alcohol (24:1), and a QIAmp PCR purification kit (QIAGEN, Hilden, Germany) [2].

### Polymerase chain reaction, cloning and sequencing

**Figure 2:**
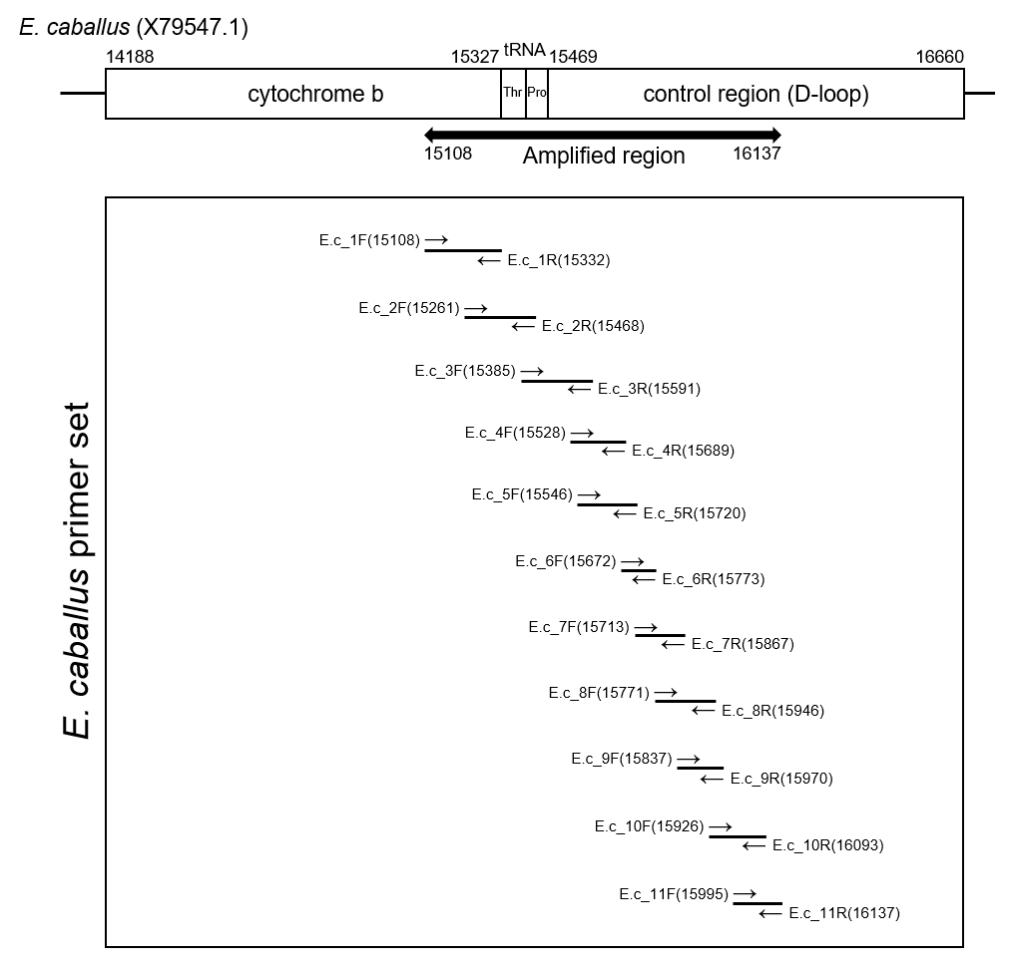
Amplification region of Equus caballus mitochondrial DNA. To recover ancient DNA sequence from Gongpyeongdong horse bone, each primer sets (E.c_1 to E.c_11) were designed to overlap the sequences of neighboring amplicon partially.

The extracted DNA (40 ng) was treated with uracil-DNA-glycosylase and subsequently mixed with AmpliTaq Gold 360 Master Mix (Thermo Fisher Scientific, Waltham, MA, USA) and primer sets (Integrated DNA Technology, Coralville, IA, USA). Polymerase chain reaction (PCR) amplification was conducted using the SimpliAmp Thermal Cycler (Applied Biosystems, Waltham, MA, USA). Figure 2 and Table 1 summarizes the data on the primers and PCR conditions, and the locations of amplicons. Amplified DNA products were separated by gel electrophoresis. PCR amplicons were then extracted using the QIAquick Gel Extraction Kit (Qiagen, Germany). Next, bacterial transformation was conducted with the pGEM-T Easy Vector system (Promega Corporation, Madison, WI, USA). Transformed bacteria were cultivated on an agar plate with ampicillin, IPTG and X-GAL. After the harvested colony was allowed to proliferate in LB media, the bacteria were purified with a QIAprep Spin Miniprep kit (Qiagen, Germany). The sequencing of the cloned DNA was conducted by the 3730xl DNA Analyzer (Applied Biosystems, USA) and with the BigDye Terminator v3.1 Cycle Sequencing Kit (Applied Biosystems, USA) [2,27]. To determine the consensus sequence, multiple alignments of clone sequences were conducted using the MEGA7 program [27]. The consensus sequence will be available in GenBank with accession number OM222617.

### Comparative study

The NCBI/BLAST tool compared the Gongpyeongdong consensus sequence with those retrieved from the GenBank database at the National Center for Biotechnology Information (NCBI). Web Browser Module and Alignment Explorer of MEGA7 program [27] were used to retrieve the DNA sequences from GenBank. The retrieved sequences were *E. caballus* mtDNA including cytochrome b (HRS 15128–15327), tRNA (HRS 15328–15468), and D-loop sequences (HRS 15469–16116) from South Korea (n = 11), Mongolia (n = 13), China (n = 11), Europe (n = 19), Middle East (n = 14), Central Asia (n = 5), and North America (n = 2). The DNA sequences of *E. przewalskii* (n = 4), *E. asinus* (n = 1), and *E. grevyi* (n = 1) also were used as outgroups. Of the retrieved taxa, the South Korean horses belonged to the modern Jeju Island breed reported by Kim et al [18] and Yoon et al [28]. With the homologous taxa of the GenBank database, multiple sequence alignments were conducted for Gongpyeongdong sequence using the ClustalW of MEGA7 program [27].

### Phylogenetic analysis

We additionally conducted a pairwise distance analysis. Base substitutions were counted per site to conjecture the evolutionary divergence between taxa. The sequences’ pairwise distance was calculated using the maximum composite likelihood model implemented in the MEGA7 program [27]. The setup of the analysis followed the protocol presented in an earlier report of ours [2]. *E. asinus* (NC_001788.1) and *E. grevyi* (NC_020432.2) were used as the outgroups. The genetic relationship of the sequences was further conjectured by means of a Phylogeny Reconstruction analysis with the MEGA7 program [27]. The taxa used in this analysis are summarized in Supplementary Table S2. The maximum likelihood (ML) method was used as well. As for the analysis setup, we again followed our previous report [2], except for the use of the Hasegawa-Kishino-Yano method for the phylogenetic tree model [27]. To determine the reliability of the constructed phylogenetic tree, bootstrapping was conducted (replication number = 500) using the MEGA7 program [2,27]. The haplogroups of *E. caballus* were estimated according to the information prescribed in Achilli et al [13], Gaunitz et al [14], and Ning et al [29].

## RESULTS AND DISCUSSION

**Figure 3:**
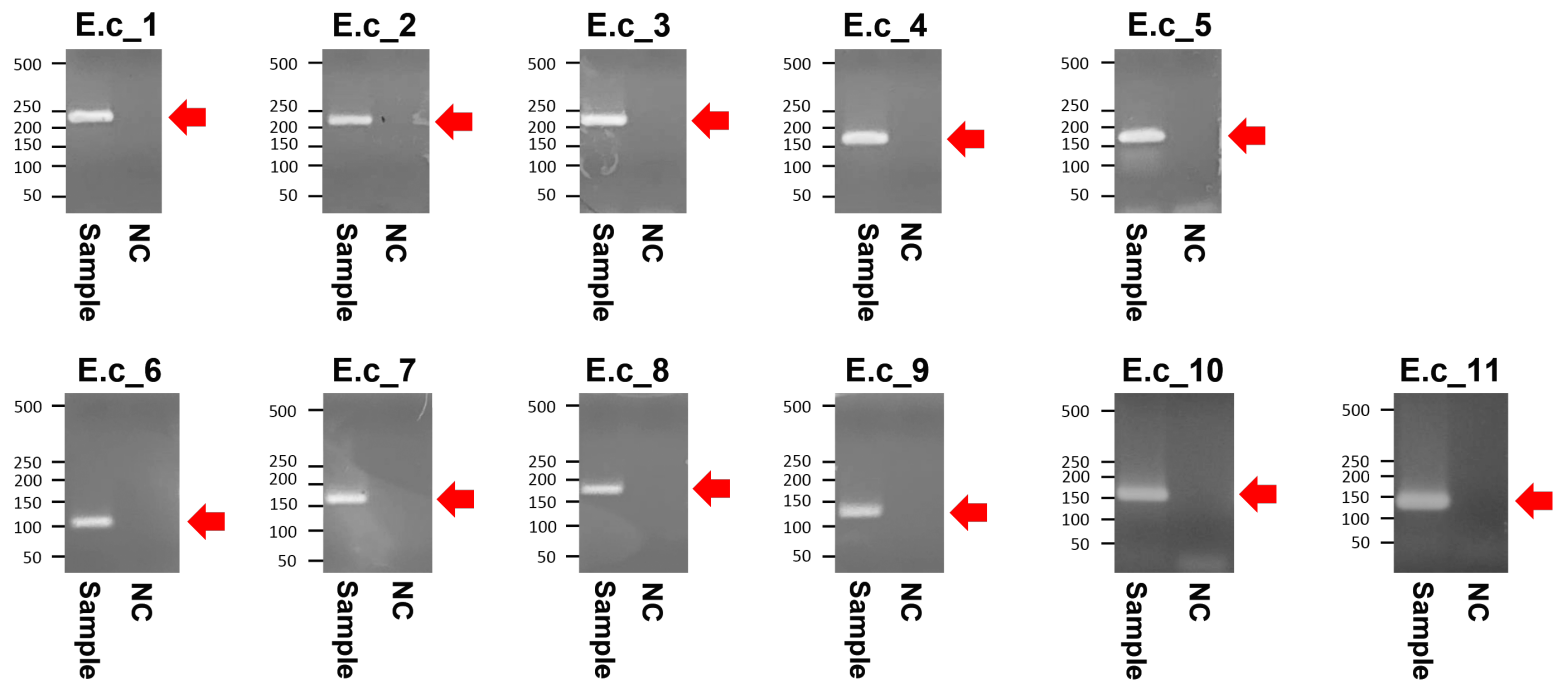
Agarose gel electrophoresis of the polymerase chain reaction amplified products from Gongpyeongdong horse bone. E.c_1 to E.c_11 represent specific amplicons (indicated by arrows) summarized in Table 1. NC, negative control.

**Figure 4:**
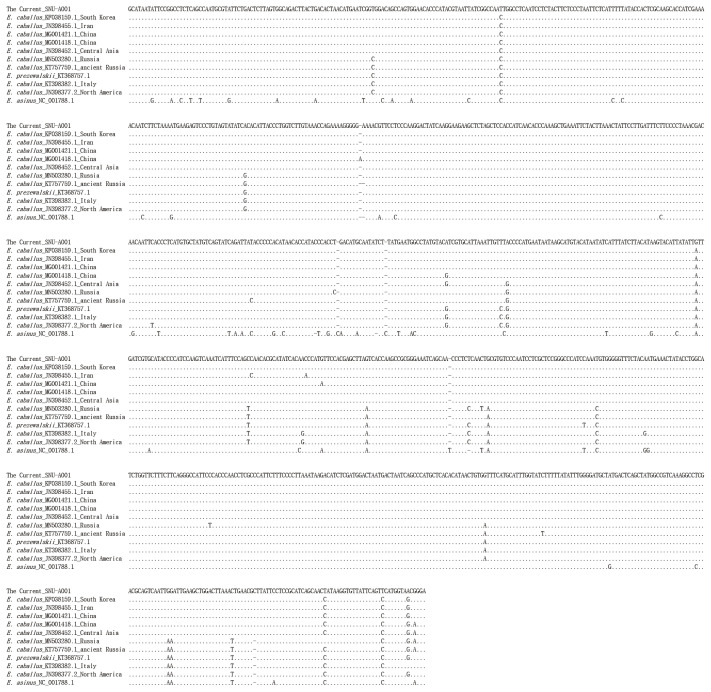
Comparison of the consensus sequence of Gongpyeongdong horse mtDNA with other sequences retrieved from GenBank.

The PCR products of the specimens under electrophoresis were shown as specific bands (11 amplicons) for the cytochrome b, tRNA, and D-loop region of *E. caballus* mtDNA, while no amplified bands were observed in the extractions of the negative controls (Figure 3). The cloned PCR products (n = 5 for each amplicon) were then sequenced and aligned to determine the consensus sequence (total length: 989 bp) (Supplementary Table S3). In NCBI/BLAST search, we could not find any taxa 100% identical to the Gongpyeongdong horse mtDNA sequence. Nevertheless, some sequences in GenBank showed remarkably high similarity (above 99%), as follows (Table 2): *E. caballus* reported from South Korea (KF038159.1, 99.49%; KF038162.1, 97.57%), Iran (JN398455.1, 99.49%), China (MG001421.1, 99.39%; MG001418.1, 99.39%; MG001419.1, 99.29%), and Central Asia (JN398452.1, 99.39%; JN398450.1, 99.09%) (Figure 4).

**Figure 5:**
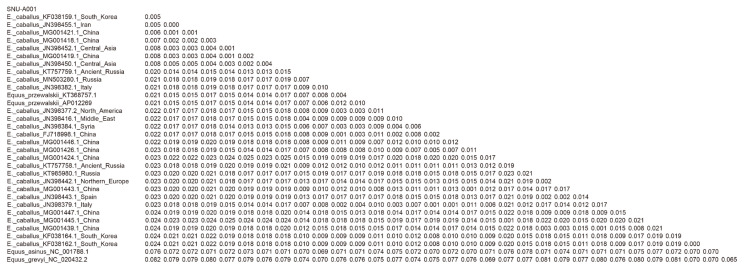
Pairwise distances between Gonpyeongdong horse and other related taxa retrieved from GenBank.

**Figure 6:**
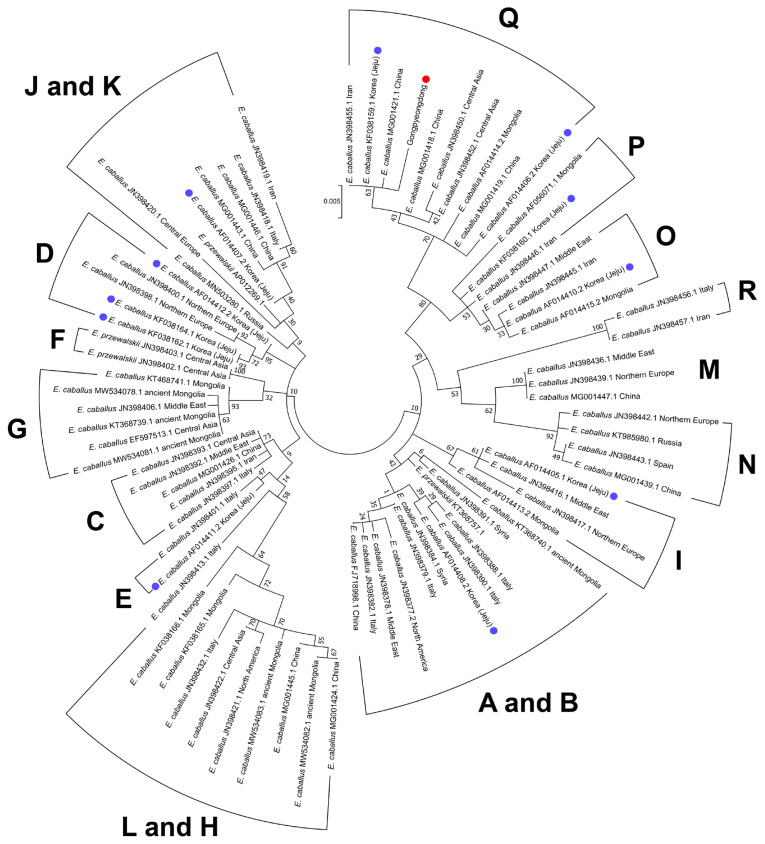
Phylogenetic analysis of the ancient horse (SNU-A001) (red dot) and the other horses retrieved from GenBank. Blue dots represent the taxa of modern Jeju horses. Numbers next to each node are bootstrap percentages. Scale shows the number of substitutions per site that are represented by a branch line of a particular length. Mitochondrial haplogroups are also marked.

As seen in Table 2, we also found that the Gongpyeong dong mitotype shares 97.97% identity with *E. przewalskii*, which is not much different from other *E. caballus* horses (ranging approximately from 97% to 99% identity). On the other hand, the Gongpyeongdong mitotype is distinct from those of the donkey (*E. asinus*; 95.94%) and imperial zebra (*E. grevyi*; 90.86%). This pattern was further confirmed by pairwise distance analysis. As seen in Figure 5, the outgroup breeds (*E. asinus* and *E. grevyi*) were genetically remote (0.076 and 0.082, respectively) from the Gongpyeongdong case, whereas the pairwise distances remarkably close to the latter were those of *E. caballus* taxa reported from South Korea (0.005), Iran (0.005), China (0.006 to 0.008), Central Asia (0.008), and other countries (Figure 5). Using mtDNA cytochrome b, tRNA, and D-loop sequences of the Gongpyeongdong case and other *E. caballus* taxa from GenBank, the phylogenetic tree could be reconstructed (Figure 6).

*E. przewalskii* horse, which inhabits the Mongolian grasslands, is morphologically distinct from modern horses, and thus has been thought to be the direct descendant of wild horse *E. ferus* [13]. However, Ishida et al [17] revealed by phylogenetic analysis that *E. przewalskii* is within the genetic variation of the Japanese domestic horse. They conjectured that the chromosome number change of *E. przewalskii* might have occurred rather recently. Another recent study revealed that *E. przewalskii* is likely a horse that was domesticated once and then returned to the wild, like the mustang of North America [14].

In Korean history, descriptions of domesticated horses are seen as early as the 2nd century BCE [3]. In the 12th century CE, Koreans determined their horses to be comprised of at least six breeds, as dependent on their body shapes [3]. However, those classifications were based only on appearance, not on any scientific evidence. In this regard, the Jeju Island horse (Jorangmal in Korean) is important to any understanding of the genetic traits of ancient or medieval Korean horses because Western horses were actively introduced starting in the 20th century, with the result that indigenous Korean horses became much rarer. Actually, many scholars consider the Jeju Island horse to represent the indigenous Korean horse populations [30].

Nevertheless, as historical and genetic research on horses proceeded, it turned out that the reality was not so simple. For instance, when Mongolians invaded the Korean Kingdom in 1276, they established ranches on Jeju Island to raise their horses. Therefore, the genetic traits of Jeju Island horses might have been seriously influenced by introgression of Mongolian breeds [3]. In fact, this conjecture was further confirmed by an mtDNA D-loop analysis exhibiting multiple origins (possibly representing Mongolian and non-Mongolian breeds) in Jeju Island horses’ maternal lineage [18]. Simply put, indigenous Korean horses have not remained the same but have been constantly changed by introgression of foreign horses in history [3,30]. This hypothesis also was supported by our phylogenetic analysis (ML method) findings. Based on the previous reports of mtDNA haplogroup using cytb and D-loop sequences [13,14,18,28,29,31–36] (Supplementary Table S4), the Gongpyeongdong horse was clustered together with the *E. caballus* taxa of haplogroup Q. According to Achilli et al [13], a horse haplogroup such as Q shows frequency peaks in Asia (13.80%) and a decline to the Middle East (10.42%) and Europe (3.85%). Ning et al [29] also confirmed that the genetic origin of haplogroup Q is likely East Asia [29]. In this sense, the present Gongpyeongdong case, the 15th century horse bones found on the mainland of Korea, can be regarded as genetically representative of the horses long established in East Asia. In the phylogenetic tree, we also noted that Jeju Island horses belong to the different clades of B, D, E, I, J, O, and Q (Figure 6). Among them, haplogroups O and Q are mostly localized to East Asia [13,29]; the ancestral haplotypes of haplogroup D, moreover, also have been localized to Asia [29]. On the other hand, the Jeju Island horse of haplogroup I looks somewhat different from those of haplogroups D, O, and Q, because the former is known to be common among Western and Southern European horses [29]. Taken together, modern Jeju Island horses include different maternal haplogroups with various genetic backgrounds. We can conjecture that various horse breeds with different genetic origins left their genetic traits among modern Korean horses.

## CONCLUSION

By obtaining a consensus mtDNA sequence from a horse bone collected at a 15th century archaeological site (Gongpyeongdong) in Old Seoul City, we confirm that the horse belongs to haplogroup Q, the genetic origin of which could be traced back to East Asia. Our data reported on the maternal lineage of Joseon period horse bones are meaningful for understanding the domestication history in East Asia, which has yet to be comprehensively revealed. We look forward to further relevant aDNA reports on ancient or medieval horse bones found at South Korean archaeological sites.

## Figures and Tables

**Table 1:** The primers and polymerase chain reaction conditions in used this study

Items	Primer	5′ to 3′	Annealing Temp. (°C)	Length (bp)
mtDNA primer set				
E.c_1	E.c-1F	CCA CAT ATC AAA ACA ACG AA	56	225
	E.c-1R	GGG ACT CTT CAT TTT AGA AGA		
E.c_2	E.c-2F	CTT CTC CCT AAT TCT CAT T	55	208
	E.c-2R	CAA GGA ATA GTT TAA GTA GAA		
E.c_3	E.c-3F	GTT TCC TCC CAA GGA CTA TC	56	207
	E.c-3R	GCA CGA TGT ACA TAG GCC ATT		
E.c_4	E.c-4F	CCC CCA CAT AAC ACC ATA CC	56	162
	E.c-4R	GAC TTG GAT GGG GTA TGC AC		
E.c_5	E.c-5F	CCC ACC TGA CAT GCA ATA TCT T	56	175
	E.c-5R	CTG TGA TAT GCG TGT TGA CTG GA		
E.c_6	E.c-6F	GCA TAC CCC ATC CAA GTC AA	55	102
	E.c-6R	TGA GAG GGT TGC TGA TTT CC		
E.c_7	E.c-7F	TAT CAC AGC CCA TGT TCC AC	56	155
	E.c-7R	ATG GCC CTG AAG AAA GAA CC		
E.c_8	E.c-8F	CCA ACT ACG TGT CCC AAT CC	55	176
	E.c-8R	GTG TGA GCA TGG GCT GAT TA		
E.c_9	E.c-9F	ACC TGG CAT CTG GTT CTT TCT	55	134
	E.c-9R	CCA AAT GCA TGA AAC CAC AG		
E.c_10	E.c-10F	CTA ATC AGC CCA TGC TCA CA	55	168
	E.c-10R	CAC CTT ATG GTT GCT GAT GC		
E.c_11	E.c-11F	ATG ACT CAG CTA TGG CCG TC	56	143
	E.c-11R	CAG GTG CAC TTG TTT CCT ATG		
PCR conditions				
Pre-denaturation		95°C; 10 min		
Denaturation		95°C; 30 s	42 cycles	
Annealing		55°C to 56°C; 30 s		
Extension		72°C; 30 s		
Final extension		72°C; 10 min		

PCR, polymerase chain reaction.

**Table 2:** BLAST searching results indicate coverage and percent identity of each taxon comparing to consensus sequence of SNU-A001 mtDNA (HRS 15128–16116)

Species	Total score (BLAST)	Coverage (%)	Percent identity (%)	Geographical region	Accession number
*E. caballus*	1,799	100	99.49	South Korea	KF038159.1
	1,799	100	99.49	Iran	JN398455.1
	1,794	100	99.39	China	MG001421.1
	1,783	99	99.39	China	MG001418.1
	1,783	99	99.39	Central Asia	JN398452.1
	1,777	99	99.29	China	MG001419.1
	1,777	100	99.09	Central Asia	JN398450.1
	1,709	99	98.07	Ancient Russia	KT757759.1
	1,703	100	97.78	China	FJ718998.1
	1,703	100	97.78	North America	JN398377.2
	1,703	99	97.97	Syria	JN398384.1
	1,703	99	97.97	Middle East	JN398416.1
	1,698	99	97.86	Italy	JN398379.1
	1,698	99	97.86	China	MG001426.1
	1,698	100	97.67	Spain	JN398443.1
	1,698	100	97.67	Northern Europe	JN398442.1
	1,698	100	97.67	Russia	KT985980.1
	1,698	100	97.67	China	MG001424.1
	1,692	100	97.57	South Korea	KF038162.1
	1,692	100	97.57	China	MG001447.1
	1,692	99	97.76	China	MG001439.1
	1,692	100	97.57	China	MG001443.1
	1,688	99	97.66	Russia	KT757740.1
*E. przewalskii*	1,705	99	97.97	Mongolia	AP012269.1
*E. asinus*	1,448	100	95.94	Worldwide (Reference sequence)	NC_001788.1
*E. grevyi*	1,408	100	90.86		NC_020432.2

GenBank accession numbers are also marked.

## SUPPLEMENTARY MATERIAL

**Supplementary Table S1**. The information on Gongpyeongdong samples discovered in layer VI.

**Supplementary Table S2**. The information of taxa used for phylogenetic analysis in Figure 6.

**Supplementary Table S3**. Alignment of cloned DNA sequences to determine the consensus sequence of the Gongpyeongdong horse mtDNA cytochrome b, tRNA, and D-loop region. E.c_1 to E.c_11 represent the primer sets in Table 1.
