# Supplementary material for: Genetic analysis of mitochondrial DNA from ancient Equus caballus bones found at archaeological site of Joseon dynasty period capital area
Source: Anim Biosci. 2022 Mar 1;35(8):1141–50. doi: 10.5713/ab.21.0500 (PMC9262724; doi:10.5713/ab.21.0500)
Supplement: Supplementary file 1 [file ab-21-0500-suppl.pdf]

Supplementary Data 1. The information on Gongpyeongdong samples discovered in layer VI.

Pit	Species	Minimum number of individuals (MNI)	Sample ID
#1	*Equus caballus*	2	SNU-A001<br>SNU-A002
#2	*Equus caballus*	1	SNU-A003
#3	*Equus caballus*	2	SNU-A004<br>SNU-A005

Supplementary Data 2. The information of taxa used for phylogenetic analysis in Figure 6.

Geographical location	Reference	Access No.	Haplogroup	Ancient Cases
Syria	Achilli et al., 2012	JN398384.1	A	
South Korea (Jeju)	Kim et al., 1999	AF014408.2		
Mongolia (ancient)	Gaunitz et al., 2018	KT368740.1		AD1842
North America	Achilli et al., 2012	JN398377.2		
Italy	Achilli et al., 2012	JN398379.1		
China	Ning et al., 2019	FJ718998.1		
Italy	Achilli et al., 2012	JN398382.1		
Denmark	Sarkissian et al., 2015	KT368757.1		
Middle East	Achilli et al., 2012	JN398378.1	B	
Italy	Achilli et al., 2012	JN398388.1		
Italy	Achilli et al., 2012	JN398390.1		
Syria	Achilli et al., 2012	JN398391.1		
China	Ma et al., 2018	MG001426.1	C	
Central Asia	Achilli et al., 2012	JN398393.1		
Middle East	Achilli et al., 2012	JN398392.1		
Iran	Achilli et al., 2012	JN398395.1		
Italy	Achilli et al., 2012	JN398397.1		
South Korea (Jeju)	Yoon et al., 2018	KF038162.1	D	
South Korea (Jeju)	Kim et al., 1999	AF014412.2		
South Korea (Jeju)	Yoon et al., 2018	KF038164.1		
Northern Europe	Achilli et al., 2012	JN398400.1		
Northern Europe	Achilli et al., 2012	JN398398.1		
South Korea (Jeju)	Kim et al., 1999	AF014411.2	E	
Italy	Achilli et al., 2012	JN398401.1		
Central Asia	Achilli et al., 2012	JN398402.1	F	
Central Asia	Achilli et al., 2012	JN398403.1		
Mongolia	Kusliy et al., 2021	MW534081.1	G	BC13-10
Mongolia	Gaunitz et al., 2018	KT368739.1		AD1843
Mongolia	Kusliy et al., 2021	MW534078.1		BC1-AD1
Mongolia	Sarkissian et al., 2015	KT368741.1		
Middle East	Achilli et al., 2012	JN398406.1		
Central Asia	GenBank	EF597513.1		
Italy	Achilli et al., 2012	JN398413.1	H	
Mongolia	Yoon et al., 2018	KF038166.1		
Middle East	Achilli et al., 2012	JN398416.1	I	
South Korea (Jeju)	Kim et al., 1999	AF014405.1		
Mongolia	Kim et al., 1999	AF014413.2		
Northern Europe	Achilli et al., 2012	JN398417.1		
North America	Goto et al., 2011	AP012269.1	JK	
China	Ma et al., 2018	MG001443.1	J	
China	Ma et al., 2018	MG001446.1		
Iran	Achilli et al., 2012	JN398419.1		
South Korea (Jeju)	Kim et al., 1999	AF014407.2		
Italy	Achilli et al., 2012	JN398418.1		
Central Europe	Achilli et al., 2012	JN398420.1		
Russia	Vershinina et al., 2020	MN503280.1	K	
Mongolia	Yoon et al., 2018	KF038165.1	L	
Mongolia (ancient)	Kusliy et al., 2021	MW534083.1		BC13-10
China	Ma et al., 2018	MG001445.1		
Mongolia (ancient)	Kusliy et al., 2021	MW534082.1		BC13-10
China	Ma et al., 2018	MG001424.1		
North America	Achilli et al., 2012	JN398421.1		
Italy	Achilli et al., 2012	JN398432.1		
Central Asia	Achilli et al., 2012	JN398422.1		

China	Ma et al., 2018	MG001447.1	M	
Middle East	Achilli et al., 2012	JN398436.1		
Northern Europe	Achilli et al., 2012	JN398439.1		
Northern Europe	Achilli et al., 2012	JN398442.1	N	
Russia	Vorobiera et al., 2020	KT985980.1		
China	Ma et al., 2018	MG001439.1		
Spain	Achilli et al., 2012	JN398443.1		
South Korea (Jeju)	Kim et al., 1999	AF014410.2	O	
Mongolia	Kim et al., 1999	AF014415.2		
Middle East	Achilli et al., 2012	JN398447.1		
Iran	Achilli et al., 2012	JN398445.1		
South Korea (Jeju)	Yoon et al., 2018	KF038160.1	P	
Mongolia	Kim et al., 1999	AF056071.1		
Iran	Achilli et al., 2012	JN398446.1		
South Korea (Jeju)	Yoon et al., 2018	KF038159.1	Q	
Iran	Achilli et al., 2012	JN398455.1		
China	Ma et al., 2018	MG001421.1		
Central Asia	Achilli et al., 2012	JN398450.1		
China	Ma et al., 2018	MG001418.1		
Central Asia	Achilli et al., 2012	JN398452.1		
China	Ma et al., 2018	MG001419.1		
Mongolia	Kim et al., 1999	AF014414.2		
South Korea (Jeju)	Kim et al., 1999	AF014406.2		
Italy	Achilli et al., 2012	JN398456.1	R	
Iran	Achilli et al., 2012	JN398457.1		

Supplementary Data 3. Alignment of cloned DNA sequences to determine the consensus sequence of the Gongpyeongdong horse mtDNA cytochrome b, tRNA, and D-loop region. E.c_1 to E.c_11 represent the primer sets in Table 1.

E.c_1

```
          1                                                                                                                                                                                      184
Consensus GCATAATATTCCGGCCTCTCAGCCAATGCGTATTCTGACTCTTAGTGGCAGACTTACTGACACTAACATGAATCGGTGGACAGCCAGTGGAACACCCATACGTAATTATCGGCCAATTGGCCTCAATCCTCTACTTCTCCCTAATTCTCATTTTTATACCACTCGCAAGCACCATCGAAAACAA
Clone 1   ........................................................................................................................................................................................
Clone 2   ........................................................................................................................................................................................
Clone 3   ........................................................................................................................................................................................
Clone 4   ........................................................................................................................................................................................
Clone 5   ........................................................................................................................................................................................
```

E.c_2

```
          1                                                                                                                                                                       167
Consensus TTTATACCACTCGCAAGCACCATCGAAAACAATCTTCTAAAATGAAGAGTCCCTGTAGTATATCACACATTACCCTGGTCTTGTAAACCAGAAAAGGGGGAAAACGTTCCTCCCAAGGACTATCAAGGAAGAAGCTCTAGCTCCACCATCAACACCCAAAGCTGAAA
Clone 1   .......................................................................................................................................................................
Clone 2   .......................................................................................................................................................................
Clone 3   .......................................................................................................................................................................
Clone 4   .......................................................................................................................................................................
Clone 5   .......................................................................................................................................................................
```

E.c_3

```
          1                                                                                                                                                                      166
Consensus AAGGAAGAAGCTCTAGCTCCACCATCAACACCCAAAGCTGAAATTCTACTTAAACTATTCCTTGATTTCTTCCCCTAAACGACAACAATTCACCCTCATGTGCTATGTCAGTATCAGATTATACCCCCACATAACACCATACCCACCTGACATGCAATATCTTATG
Clone 1   ......................................................................................................................................................................
Clone 2   ......................................................................................................................................................................
Clone 3   .............................................................................................T........................................................................
Clone 4   ......................................................................................................................................................................
Clone 5   .............................................................................................T........................................................................
```

E.c_4

```
          1                                                                                                                      122
Consensus CACCTGACATGCAATATCTTATGAATGGCCTATGTACATCGTGCATTAAATTGTTTACCCCATGAATAATAAGCATGTACATAATATCATTTATCTTACATAAGTACATTATATTGTTGATC
Clone 1   ..........................................................................................................................
Clone 2   ...................................................................................................................A......
Clone 3   ..........................................................................................................................
Clone 4   ..........................................................................................................................
Clone 5   ...................................................................................................................A......
```

E.c_5

```
          1                                                                                                                              130
Consensus ATGAATGGCCTATGTACATCGTGCATTAAATTGTTTACCCCATGAATAATAAGCATGTACATAATATCATTTATCTTACATAAGTACATTATATTGTTGATCGTGCATACCCCATCCAAGTCAAATCATT
Clone 1   ..................................................................................................................................
Clone 2   ..................................................................................................................................
Clone 3   ..................................................................................................................................
Clone 4   ..................................................................................................................................
Clone 5   ..................................................................................................................................
```

E.c_6

```
          1                                                            62
Consensus ATCATTTCCAGCCAACACGCATATCACAACCCATGTTCCACGAGCTTAGTCACCAAGCCGCG
Clone 1   ..............................................................
Clone 2   ..............................................................
Clone 3   ............................................T.................
Clone 4   ..............................................................
Clone 5   ..............................................................
```

E.c_7

```
          1                                                                                                               115
Consensus GAGCTTAGTCACCAAGCCGCGGGAAATCAGCAACCCTCTCAACTGCGTGTCCCAATCCTCGCTCCGGGCCCATCCAAATGTGGGGGTTTCTACAATGAAACTATACCTGGCATCT
Clone 1   ...................................................................................................................
Clone 2   ...................................................................................................................
Clone 3   ................................................................................................A..................
Clone 4   ...................................................................................................................
Clone 5   ...................................................................................................................
```

E.c_8

```
          1                                                                                                                                    136
Consensus TCGCTCCGGGCCCATCCAAATGTGGGGGTTTCTACAATGAAACTATACCTGGCATCTGGTTCTTTCTTCAGGGCCATTCCCACCCAACCTCGCCCATTCTTTCCCCTTAAATAAGACATCTCGATGGACTAATGAC
Clone 1   ........................................................................................................................................
Clone 2   ........................................................................................................................................
Clone 3   ........................................................................................................................................
Clone 4   ........................................................................................................................................
Clone 5   ........................................................................................................................................
```

E.c_9

```
          1                                                                                             93
Consensus TCAGGGCCATTCCCACCCAACCTCGCCCATTCTTTCCCCTTAAATAAGACATCTCGATGGACTAATGACTAATCAGCCCATGCTCACACATAA
Clone 1   .............................................................................................
Clone 2   .............................................................................................
Clone 3   .............................................................................................
Clone 4   .............................................................................................
Clone 5   .................................................................A...........................
```

E.c_10

```
          1                                                                                                                             129
Consensus CATAACTGTGGTTTCATGCATTTGGTATCTTTTTATATTTGGGGATGCTATGACTCAGCTATGGCCGTCAAAGGCCTCGACGCAGTCAATTGGATTGAAGCTGGACTTAAACTGAACGCTTATTCCTCC
Clone 1   .................................................................................................................................
Clone 2   .................................................................................................................................
Clone 3   .................................................................................................................................
Clone 4   .................................................................................................................................
Clone 5   .................................................................................................................................
```

E.c_11

```
          1                                                                                                     103
Consensus AAAGGCCTCGACGCAGTCAATTGGATTGAAGCTGGACTTAAACTGAACGCTTATTCCTCCGCATCAGCAACTATAAGGTGTTATTCAGTTCATGGTAACGGGA
Clone 1   .......................................................................................................
Clone 2   .......................................................................................................
Clone 3   .......................................................................................................
Clone 4   .......................................................................................................
Clone 5   .......................................................................................................
```

Supplementary Data 4. The haplotypes and nucleotide substitutions including Gongpyeongdong sample (SNU-A0001). All the species of Supplementary Data 2 were used for alignment. The partial cytb, complete tRNA(Thr) and tRNA(Pro), and partial control region (D-loop) (HRS 15128 - 16116) alignment result shows that 71 polymorphic sites are present in Equus spp. belonging to 18 haplogroup (A to R). MtDNA sequences were aligned using MEGA 7 software.

			cytochrome b						tRNA-Thr			control region (D-loop)																										
No.	Haplogroup		15135	15144	15204	15249	15252	15315	15344	15386	15388	15494	15495	15496	15521	15526	15532	15534	15538	15540	15542	15585	15595	15597	15598	15601	15602	15603	15604	15615	15616	15617	15635	15649	15650	15659	15666	15667
		E caballus X79547.1 (Reference sequence, HRS)	A	T	C	C	A	T	G	T	T	T	T	A	G	T	C	C	A	A	C	G	A	A	T	T	C	T	G	A	A	T	C	A	A	T	G	A
1	A	E caballus JN398384.1 Syria	.	.	.	.	.	.	.	.	-	.	C	.	.	.	.	.	.	.	.	.	.	.	.	.	.	.	.	.	.	.	.	.	.	.	.	.
2	A	E caballus AF014408.2 Korea Jeju								.	-	.	C	.	.	.	.	.	.	.	.	.	.	.	.	.	.	.	.	.	.	.	.	.	G	.	A	.
3	A	E caballus KT368740.1 Mongolia ancient	.	.	.	.	.	.	.	.	-	.	C	.	.	.	.	.	.	.	.	A	.	.	.	.	.	C	.	.	.	.	.	.	.	.	.	.
4	A	E caballus JN398377.2 North America	.	.	.	.	.	.	.	.	-	.	.	.	.	.	.	.	.	.	.	.	.	.	.	.	.	.	.	.	.	.	.	.	.	.	.	.
5	A	E caballus JN398379.1 Italy	.	.	.	.	.	.	.	.	-	.	C	.	.	.	.	.	.	.	.	.	.	.	.	.	.	.	.	.	.	.	.	.	.	.	.	.
6	A	E caballus FJ718998.1 China	.	.	.	.	.	.	.	.	-	.	C	.	.	.	.	.	.	.	.	.	.	.	.	.	.	.	.	.	.	.	.	.	.	.	.	.
7	A	E caballus JN398382.1 Italy	.	.	.	.	.	.	.	.	-	.	C	.	.	.	.	.	.	.	.	.	.	.	.	.	.	.	.	.	.	.	.	.	.	.	.	.
8	A	E przewalskii KT368757.1 Denmark	.	.	.	.	.	.	.	.	-	.	C	.	.	.	.	.	.	.	.	.	.	.	.	.	.	.	.	.	.	.	.	.	.	.	.	.
9	B	E caballus JN398378.1 Middle East	.	.	.	.	.	.	.	.	-	.	C	.	.	.	.	.	.	.	.	A	.	.	.	.	.	.	.	.	.	.	.	.	.	.	.	.
10	B	E caballus JN398388.1 Italy	.	.	.	.	.	.	.	.	-	.	C	.	.	.	.	.	.	.	.	A	.	.	.	.	.	.	.	.	.	.	.	.	G	.	A	.
11	B	E caballus JN398390.1 Italy	.	.	.	.	.	.	.	.	-	.	C	.	.	.	.	.	.	.	.	.	.	.	.	.	.	.	.	.	.	.	.	.	G	.	A	.
12	B	E caballus JN398391.1 Syria	.	.	.	.	.	.	.	.	-	.	C	.	.	.	.	.	.	.	.	.	.	.	.	.	.	.	.	.	.	.	.	.	G	.	.	.
13	C	E caballus MG001426.1 China	.	.	.	.	.	.	.	-	.	.	C	.	.	.	.	.	.	.	.	.	.	.	.	.	T	.	.	.	.	.	.	.	G	.	.	.
14	C	E caballus JN398392.1 Middle East	.	.	.	.	.	.	.	-	.	.	C	.	.	.	.	.	.	.	.	.	.	.	.	.	T	.	.	.	.	.	.	.	G	.	.	.
15	C	E caballus JN398393.1 Central Asia	.	.	.	.	.	.	.	-	.	.	C	.	.	.	.	.	.	.	.	.	.	.	.	.	T	.	.	.	.	.	.	.	G	.	.	.
16	C	E caballus JN398395.1 Iran	.	.	.	.	.	.	.	-	.	.	C	.	.	.	.	.	.	.	.	.	.	.	.	.	T	.	.	.	.	.	.	.	G	.	.	.
17	C	E caballus JN398397.1 Italy	.	.	.	.	.	.	.	-	.	.	C	.	.	.	.	.	.	.	.	.	.	G	.	.	T	.	.	.	.	.	.	.	G	.	.	.
18	D	E caballus KF038162.1 Korea Jeju	.	.	.	.	.	.	.	.	-	.	C	.	A	.	.	.	.	.	.	.	.	.	.	.	T	.	.	.	.	C	.	.	.	.	.	.
19	D	E caballus AF014412.2 Korea Jeju								.	-	.	C	.	.	.	.	.	.	.	.	.	.	.	.	.	T	.	.	.	.	.	.	.	.	.	.	.
20	D	E caballus KF038164.1 Korea Jeju	.	.	.	.	.	.	.	.	-	.	C	.	A	.	.	.	.	.	.	.	.	.	.	.	T	.	.	.	.	C	.	.	.	.	.	.
21	D	E caballus JN398400.1 Northern Europe	.	.	.	.	.	.	.	.	-	.	C	.	A	.	.	.	.	.	.	.	.	.	.	.	T	.	.	.	.	.	.	.	.	.	.	.
22	D	E caballus JN398398.1 Northern Europe	.	.	.	.	.	.	.	.	-	.	C	.	A	.	.	.	.	.	.	.	.	.	.	.	T	.	.	.	.	.	.	.	.	.	.	.
23	E	E caballus AF014411.2 Korea Jeju								-	.	.	C	.	.	.	.	.	.	.	.	A	.	.	.	.	T	.	.	.	.	.	.	.	G	.	A	.
24	E	E caballus JN398401.1 Italy	.	.	.	.	.	.	.	-	.	.	C	.	.	.	.	.	.	.	T	A	.	G	.	.	T	.	.	.	.	.	.	.	G	.	A	.
25	F	E przewalskii JN398402.1 Central Asia	.	.	.	.	.	.	.	-	.	.	C	.	.	.	.	.	.	.	T	.	G	.	.	.	T	.	.	.	.	.	.	.	G	.	A	.
26	F	E przewalskii JN398403.1 Central Asia	.	.	.	.	.	.	.	-	.	.	C	.	.	.	.	.	.	.	T	.	G	.	.	.	T	.	.	.	.	.	.	.	G	.	A	.
27	G	E caballus MW534081.1 Mongolia ancient	.	.	.	.	.	.	.	-	.	.	C	.	.	.	.	.	.	.	T	A	.	G	.	.	T	.	.	.	.	.	T	.	G	.	A	.
28	G	E caballus KT368739.1 Mongolia ancient	.	.	.	.	.	.	.	-	.	.	C	.	.	.	.	.	.	.	T	A	.	G	.	.	T	.	.	.	.	.	T	.	G	.	A	.
29	G	E caballus MW534078.1 Mongolia ancient	.	.	.	.	.	.	.	-	.	.	C	.	.	.	.	.	.	.	T	A	.	G	.	.	T	.	.	.	.	.	Y	.	R	.	A	.
30	G	E caballus KT368741.1 Mongolia	.	.	.	.	.	.	.	-	.	.	C	.	.	.	.	.	.	.	T	A	.	G	.	.	T	.	.	.	.	.	T	.	G	.	A	.
31	G	E caballus JN398406.1 Middle East	.	.	.	.	.	.	.	-	.	.	C	.	.	.	.	.	.	.	T	A	.	G	.	.	T	.	.	G	.	.	T	.	G	.	A	.
32	G	E caballus EF597513.1 Central Asia	.	.	.	.	.	.	.	-	.	.	C	.	.	.	.	.	.	.	T	A	.	G	.	.	T	.	.	.	.	.	T	.	G	.	A	.
33	H	E caballus JN398413.1 Italy	.	.	.	.	.	.	.	-	.	.	C	.	.	C	.	.	.	G	.	A	.	.	.	.	T	.	.	.	.	.	.	G	.	.	.	.
34	H	E caballus KF038166.1 Mongolia	.	.	.	.	.	C	.	-	.	.	.	.	.	C	.	T	.	G	.	A	.	.	.	.	T	.	.	.	.	.	.	G	.	.	.	.
35	I	E caballus JN398416.1 Middle East	.	.	.	.	.	.	.	-	.	.	C	.	.	.	.	.	G	.	.	A	.	.	.	.	T	.	.	.	.	.	.	.	.	.	.	.
36	I	E caballus AF014405.1 Korea Jeju								-	.	.	C	.	.	.	.	.	G	.	.	A	.	.	.	.	T	.	.	.	.	.	.	.	.	.	.	.
37	I	E caballus AF014413.2 Mongolia								-	.	.	C	.	.	.	.	.	G	.	.	.	.	G	.	.	T	.	.	.	.	.	.	.	G	.	.	.
38	I	E caballus JN398417.1 Northern Europe	.	.	.	.	.	.	.	-	.	.	C	.	.	.	.	.	G	.	.	A	.	.	.	.	T	.	.	.	.	.	.	.	G	.	.	.
39	JK	E przewalskii AP012269.1 North America	.	.	.	.	.	.	.	N	.	.	C	.	.	.	.	.	.	.	.	A	.	.	.	.	T	.	.	.	.	.	.	.	.	.	.	.
40	J	E caballus MG001443.1 China	.	.	.	.	.	.	.	-	.	.	C	.	.	.	-	.	.	.	.	A	.	.	.	.	T	.	.	.	.	.	.	.	.	.	.	.
41	J	E caballus MG001446.1 China	.	.	.	.	.	.	.	-	.	.	C	.	.	.	-	.	.	.	.	A	.	.	.	.	T	.	.	.	.	.	.	.	.	.	.	.
42	J	E caballus JN398419.1 Iran	.	.	.	.	.	.	.	-	.	.	C	.	.	.	-	.	.	.	.	A	.	.	.	.	T	.	A	.	.	.	.	.	G	.	.	.
43	J	E caballus AF014407.2 Korea Jeju								-	.	.	C	.	.	.	-	.	.	.	.	A	.	.	.	.	T	.	.	.	.	.	.	.	.	.	.	.
44	J	E caballus JN398418.1 Italy	.	.	.	.	.	.	.	-	.	.	C	.	.	.	-	.	.	.	.	A	.	.	.	.	T	.	A	.	.	.	.	.	.	.	.	.
45	J	E caballus JN398420.1 Central Europe	.	.	.	.	.	.	.	-	.	.	C	.	.	.	.	.	.	.	.	.	.	.	.	.	T	.	.	.	.	.	T	.	G	.	.	.
46	K	E caballus MN503280.1 Russia	.	.	.	.	.	.	.	-	.	.	C	.	.	.	.	.	.	.	.	A	.	.	.	.	T	.	.	.	.	.	.	.	.	.	.	.
47	L	E caballus KF038165.1 Mongolia	.	.	.	.	.	C	.	-	.	.	.	.	.	.	.	T	.	.	.	A	.	.	.	.	.	C	.	.	.	.	.	G	.	.	.	.
48	L	E caballus MW534083.1 Mongolia ancient	.	.	.	.	.	C	.	-	.	C	C	G	.	.	.	T	.	.	.	A	.	.	.	.	.	C	.	.	.	.	.	G	.	.	.	.
49	L	E caballus MG001445.1 China	.	.	.	.	.	C	.	-	.	C	C	G	.	.	.	T	.	.	.	A	.	.	.	.	T	C	.	.	.	.	.	G	.	.	.	.
50	L	E caballus MW534082.1 Mongolia ancient	.	.	.	.	.	C	.	-	.	C	C	G	.	.	.	T	.	.	.	A	.	.	.	.	T	C	A	.	.	.	.	G	.	.	.	.
51	L	E caballus MG001424.1 China	.	.	.	.	.	C	.	-	.	C	C	G	.	.	.	T	.	.	.	A	.	.	.	.	T	C	A	.	.	.	.	G	.	.	.	.
52	L	E caballus JN398421.1 North America	.	.	.	.	.	C	.	-	.	C	C	G	.	.	.	T	.	.	.	.	.	.	.	.	T	C	.	.	.	.	.	G	.	.	.	.
53	L	E caballus JN398432.1 Italy	.	.	.	.	.	C	.	-	.	C	C	G	.	.	.	T	.	.	.	A	.	.	.	.	T	C	A	.	.	.	.	G	.	.	.	.
54	L	E caballus JN398422.1 Central Asia	.	.	.	.	.	C	.	-	.	C	C	G	.	.	.	T	.	.	.	.	.	.	.	.	T	C	.	.	.	.	.	G	.	.	.	.
55	M	E caballus MG001447.1 China	G	.	.	.	.	.	A	-	.	.	C	.	.	.	.	.	.	.	.	.	.	.	.	.	T	.	.	.	.	C	.	.	.	C	.	.
56	M	E caballus JN398436.1 Middle East	G	.	.	.	.	.	A	-	.	.	C	.	.	.	.	.	.	.	.	.	.	.	.	.	T	.	.	.	.	C	.	.	.	C	.	.
57	M	E caballus JN398439.1 Northern Europe	G	.	.	.	.	.	A	-	.	.	C	.	.	.	.	.	.	.	.	.	.	.	.	.	T	.	.	.	.	C	.	.	.	C	.	.
58	N	E caballus JN398442.1 Northern Europe	G	.	.	.	.	.	A	-	.	.	C	.	.	.	.	.	.	.	.	.	.	.	.	C	T	.	.	.	.	.	.	.	.	.	.	.
59	N	E caballus KT985980.1 Russia	G	.	.	.	.	.	A	-	.	.	C	.	.	.	.	.	.	.	.	.	.	.	.	C	T	.	.	.	.	.	.	.	.	.	.	.
60	N	E caballus MG001439.1 China	G	.	.	.	.	.	A	-	.	.	C	.	.	.	.	.	.	.	.	A	.	.	.	C	T	.	.	.	.	.	.	.	.	.	.	.
61	N	E caballus JN398443.1 Spain	G	.	.	.	.	.	A	-	.	.	C	.	.	.	.	.	.	.	.	A	.	.	.	C	T	.	.	.	.	.	.	.	.	.	.	.
62	O	E caballus AF014410.2 Korea Jeju								-	.	.	C	.	.	.	.	.	.	.	.	.	.	G	.	.	T	.	A	.	.	.	.	.	.	.	.	G
63	O	E caballus AF014415.2 Mongolia								-	.	.	C	.	.	.	.	.	.	.	.	.	.	G	.	.	T	.	A	.	.	.	.	.	.	.	.	G
64	O	E caballus JN398447.1 Middle East	.	.	T	.	.	.	A	-	.	.	C	.	.	.	.	.	.	.	.	.	.	G	.	.	T	.	.	.	.	.	.	.	.	.	.	G
65	O	E caballus JN398445.1 Iran	.	.	T	.	.	.	A	-	.	.	C	.	.	.	.	.	.	.	.	.	.	G	.	.	T	.	A	.	.	.	T	.	.	.	.	G
66	P	E caballus KF038160.1 Korea Jeju	.	.	T	.	.	.	A	-	.	.	C	.	.	.	.	.	.	.	.	.	.	.	.	.	.	.	.	.	.	.	.	.	.	.	.	G
67	P	E caballus AF056071.1 Mongolia							N	-	.	.	C	.	.	.	.	.	.	.	.	.	.	G	.	.	T	.	A	.	.	.	.	G	.	.	.	G
68	P	E caballus JN398446.1 Iran	.	.	T	.	.	.	A	-	.	.	C	.	.	.	.	.	.	.	.	.	.	.	.	.	T	.	.	.	.	.	.	.	.	.	.	G
69	Q	E caballus OM222617.1 Korea Gongpyeongdong ancient	.	.	T	.	.	.	A	-	.	.	C	.	.	.	.	.	.	.	.	A	.	.	.	.	T	.	A	.	.	.	.	.	.	.	.	.
70	Q	E caballus KF038159.1 Korea Jeju	.	.	T	.	.	.	A	-	.	.	C	.	.	.	.	.	.	.	.	A	.	.	.	.	T	.	A	.	.	.	.	.	.	.	.	.
71	Q	E caballus JN398455.1 Iran	.	.	T	.	.	.	A	-	.	.	C	.	.	.	.	.	.	.	.	A	.	.	.	.	T	.	A	.	.	.	.	.	.	.	.	.
72	Q	E caballus MG001421.1 China	.	.	T	.	.	.	A	-	.	.	C	.	.	.	.	.	.	.	.	A	.	.	.	.	T	.	A	.	.	.	.	.	.	.	.	.
73	Q	E caballus JN398450.1 Central Asia	.	.	T	.	.	.	A	-	.	.	C	.	.	.	.	.	.	.	.	.	.	.	.	.	T	.	.	.	.	.	.	.	.	.	.	.
74	Q	E caballus MG001418.1 China	.	.	T	.	.	.	A	-	.	.	C	.	.	.	.	.	.	.	.	.	.	.	.	.	T	.	A	.	.	.	.	.	.	.	.	.
75	Q	E caballus JN398452.1 Central Asia	.	.	T	.	.	.	A	-	.	.	C	.	.	.	.	.	.	.	.	.	.	.	.	.	T	.	.	.	.	.	.	.	.	.	.	.
76	Q	E caballus MG001419.1 China	.	.	T	.	.	.	A	-	.	.	C	.	.	.	.	.	.	.	.	.	.	.	.	.	T	.	A	.	.	.	.	.	.	.	.	.
77	Q	E caballus AF014414.2 Mongolia								-	.	.	C	.	.	.	.	.	.	.	.	.	G	G	.	.	T	.	A	.	.	.	.	.	.	.	.	G
78	Q	E caballus AF014406.2 Korea Jeju								-	.	.	C	.	.	.	.	.	.	.	.	A	.	.	.	.	T	.	A	.	.	.	.	.	G	.	.	.
79	R	E caballus JN398456.1 Italy	.	C	.	T	T	.	.	-	.	.	C	.	.	.	.	.	.	.	.	.	.	.	C	.	T	.	.	G	G	.	.	.	.	C	.	.
80	R	E caballus JN398457.1 Iran	.	C	.	T	T	.	.	-	.	.	C	.	.	.	.	.	.	.	.	.	.	.	C	.	T	.	.	G	G	.	.	.	.	C	.	.

No.	Haplogroup		control region (D-loop)																																	
			15703	15709	15720	15737	15740	15770	15771	15775	15776	15777	15806	15807	15809	15811	15826	15827	15838	15868	15869	15870	15871	15956	15974	15996	16007	16031	16037	16038	16055	16057	16063+	16068	16071	16080	16081
		E caballus X79547.1 (Reference sequence, HRS)	T	C	G	T	A	C	C	C	T	A	C	C	A	C	A	A	C	T	C	C	C	A	C	T	T	T	A	A	A	T	-	T	T	G	C
1	A	E caballus JN398384.1 Syria	.	.	A	.	.	.	.	.	.	.	.	.	.	.	G	.	.	.	.	.	.	.	.	.	.	.	.	.	.	.	-	.	.	.	.
2	A	E caballus AF014408.2 Korea Jeju	.	.	A	.	.	.	.	.	.	.	.	.	.	.	G	.	.	.	.	.	.	.	T	.	.	.	.	.	.	.	-	.	.	.	.
3	A	E caballus KT368740.1 Mongolia ancient	.	.	A	.	.	.	.	.	.	.	.	.	.	.	G	.	.	.	.	.	.	.	.	.	.	.	G	.	.	.	-	.	.	.	.
4	A	E caballus JN398377.2 North America	.	.	.	.	.	.	.	.	.	.	.	.	.	.	.	.	.	.	.	.	.	.	.	.	.	.	.	.	.	.	-	.	.	.	.
5	A	E caballus JN398379.1 Italy	.	.	.	.	.	.	.	.	.	.	.	.	.	.	G	.	.	.	.	.	.	.	.	.	.	.	.	.	.	.	-	.	.	.	.
6	A	E caballus FJ718998.1 China	.	.	.	.	.	.	.	.	.	.	.	.	.	.	G	.	.	.	.	.	.	.	.	.	.	.	.	.	.	.	-	.	.	.	.
7	A	E caballus JN398382.1 Italy	.	.	.	.	.	.	.	.	.	.	.	.	.	.	G	.	.	.	.	.	.	.	.	.	.	.	.	.	.	.	-	.	.	.	.
8	A	E przewalskii KT368757.1 Denmark	.	.	A	.	.	.	.	.	.	.	.	T	.	.	.	.	.	.	.	.	.	.	.	.	.	.	.	.	.	.	-	.	.	.	.
9	B	E caballus JN398378.1 Middle East	.	.	.	.	.	.	.	.	.	.	.	.	.	.	G	.	.	.	.	.	.	.	.	.	.	.	.	.	.	.	-	.	.	.	.
10	B	E caballus JN398388.1 Italy	.	.	A	.	.	.	.	.	.	.	.	.	.	.	G	.	.	.	.	.	.	.	.	.	.	.	.	.	G	.	-	.	.	.	.
11	B	E caballus JN398390.1 Italy	.	.	A	.	.	.	.	.	.	.	.	.	.	.	G	.	.	.	.	.	.	.	.	.	.	.	.	.	G	.	-	.	.	.	.
12	B	E caballus JN398391.1 Syria	.	.	A	.	.	.	.	.	.	.	.	.	.	.	.	.	.	.	.	.	.	.	.	.	.	.	.	.	G	.	-	.	.	.	.
13	C	E caballus MG001426.1 China	.	.	A	.	.	.	.	.	.	.	.	.	.	.	G	.	.	.	.	T	.	G	T	.	.	.	.	.	.	.	-	.	.	.	.
14	C	E caballus JN398392.1 Middle East	.	.	A	.	.	.	.	.	.	.	.	.	.	.	G	.	.	.	.	T	.	G	T	.	.	.	.	.	.	.	-	.	.	.	.
15	C	E caballus JN398393.1 Central Asia	.	.	A	.	.	.	.	.	.	.	.	.	.	.	G	.	.	.	.	T	.	G	T	.	.	.	.	.	.	.	-	.	.	.	.
16	C	E caballus JN398395.1 Iran	.	.	A	.	.	.	.	.	.	.	.	.	.	.	G	.	.	.	.	T	.	G	T	.	C	.	.	.	.	.	-	.	.	.	.
17	C	E caballus JN398397.1 Italy	.	.	A	.	.	.	.	.	.	.	.	.	.	.	G	.	.	.	.	T	.	G	T	.	.	.	.	.	.	.	-	.	.	.	.
18	D	E caballus KF038162.1 Korea Jeju	.	.	A	C	.	T	.	.	.	.	.	.	.	.	.	.	.	.	.	T	.	.	.	.	.	.	.	.	.	.	-	.	.	.	.
19	D	E caballus AF014412.2 Korea Jeju	.	.	A	C	.	T	.	.	.	.	.	.	.	.	.	.	.	.	.	T	.	.	.	.	.	.	.	.	.	.	-	.	.	.	.
20	D	E caballus KF038164.1 Korea Jeju	.	.	A	C	.	T	.	.	.	.	.	.	.	.	.	.	.	.	.	T	.	.	.	.	.	.	.	.	.	.	-	.	.	.	.
21	D	E caballus JN398400.1 Northern Europe	.	.	A	C	.	T	.	.	.	.	.	.	.	.	.	.	.	.	.	T	.	.	.	.	.	.	.	.	.	.	-	.	.	.	.
22	D	E caballus JN398398.1 Northern Europe	.	.	A	C	.	T	.	.	.	.	.	.	.	.	.	.	.	C	.	T	.	.	.	.	.	.	.	.	.	.	-	.	.	.	.
23	E	E caballus AF014411.2 Korea Jeju	.	.	A	.	.	.	T	.	.	.	.	.	.	.	.	.	.	.	.	T	.	G	T	.	.	.	.	.	.	.	-	.	.	.	.
24	E	E caballus JN398401.1 Italy	.	.	A	.	.	.	T	.	.	.	.	.	.	.	.	.	.	.	.	T	.	G	T	.	.	.	.	.	.	.	-	.	.	.	.
25	F	E przewalskii JN398402.1 Central Asia	.	.	A	.	.	.	.	.	.	.	.	.	.	.	.	.	.	C	.	T	.	.	.	.	.	.	.	.	.	.	-	.	.	.	.
26	F	E przewalskii JN398403.1 Central Asia	.	.	A	.	.	.	.	.	.	.	.	.	.	.	.	.	.	C	.	T	.	.	.	.	.	.	.	.	.	.	-	.	.	.	.
27	G	E caballus MW534081.1 Mongolia ancient	C	.	A	.	.	.	.	.	.	.	.	.	.	.	.	.	.	.	.	T	.	.	.	.	.	C	.	.	.	.	-	.	.	.	.
28	G	E caballus KT368739.1 Mongolia ancient	C	.	A	.	.	.	.	.	.	.	.	.	.	.	.	.	.	.	.	T	.	.	.	.	.	C	.	.	.	.	-	.	.	.	.
29	G	E caballus MW534078.1 Mongolia ancient	C	.	A	.	.	.	.	.	.	.	T	.	.	.	.	.	.	.	.	T	.	.	.	.	.	C	.	.	.	.	-	.	.	.	.
30	G	E caballus KT368741.1 Mongolia	C	.	A	.	.	.	.	.	.	.	.	.	.	.	.	.	.	.	.	T	.	.	.	.	.	.	.	.	.	.	-	.	.	.	.
31	G	E caballus JN398406.1 Middle East	C	.	A	.	.	.	.	.	.	.	.	.	.	.	.	.	.	.	.	T	.	.	.	.	.	C	.	.	.	.	-	.	.	.	.
32	G	E caballus EF597513.1 Central Asia	C	.	A	.	.	.	.	.	.	.	.	.	.	.	.	.	.	.	.	T	.	.	.	.	.	C	.	.	.	.	-	.	.	.	.
33	H	E caballus JN398413.1 Italy	.	.	A	.	.	.	T	.	.	.	T	.	.	.	.	.	.	.	.	T	.	.	T	.	.	.	.	.	.	.	-	.	.	.	T
34	H	E caballus KF038166.1 Mongolia	.	.	A	.	.	.	T	.	.	.	.	.	.	.	.	.	.	.	.	T	T	G	T	.	.	.	.	.	.	.	-	C	.	A	T
35	I	E caballus JN398416.1 Middle East	.	T	A	.	.	.	T	.	.	.	.	.	.	.	G	.	.	.	.	.	.	.	T	.	.	.	.	.	.	.	-	.	.	.	.
36	I	E caballus AF014405.1 Korea Jeju	.	T	A	.	.	.	T	.	.	.	.	.	.	.	G	.	.	.	.	.	.	.	T	.	.	.	.	.	.	.	-	.	.	.	.
37	I	E caballus AF014413.2 Mongolia	.	T	A	.	.	.	T	.	.	.	.	.	.	.	G	.	.	.	.	.	.	.	.	.	.	.	.	.	.	.	-	.	.	.	.
38	I	E caballus JN398417.1 Northern Europe	.	T	A	.	.	.	T	.	.	.	.	.	.	.	G	.	.	.	.	T	.	.	T	.	.	.	.	.	.	.	-	.	.	A	.
39	JK	E przewalskii AP012269.1 North America	.	.	A	.	.	.	T	T	.	.	.	.	.	.	.	.	.	.	.	T	T	.	.	.	.	.	G	.	.	.	-	.	.	.	.
40	J	E caballus MG001443.1 China	.	.	A	.	.	.	T	.	.	.	.	T	.	.	.	G	.	.	.	T	T	.	.	.	.	.	.	.	.	.	-	.	C	.	.
41	J	E caballus MG001446.1 China	.	.	A	.	.	.	T	.	.	.	.	T	.	.	.	G	.	.	.	T	T	.	.	.	.	.	.	.	.	.	-	.	C	.	.
42	J	E caballus JN398419.1 Iran	.	.	A	.	.	.	T	.	.	.	.	T	.	.	.	G	.	.	.	T	T	.	.	.	.	.	.	.	.	.	-	.	C	.	.
43	J	E caballus AF014407.2 Korea Jeju	.	.	A	.	.	.	T	.	.	.	.	.	.	.	.	.	.	.	.	T	T	.	.	.	.	.	.	.	.	.	-	.	.	.	.
44	J	E caballus JN398418.1 Italy	.	.	A	.	.	.	T	.	.	.	.	T	.	.	.	G	.	.	.	T	T	.	.	.	.	.	.	.	.	.	-	.	C	.	.
45	J	E caballus JN398420.1 Central Europe	.	.	A	.	.	.	T	T	.	.	.	T	.	.	.	.	.	.	.	T	T	G	.	.	.	.	.	.	.	.	-	.	C	.	.
46	K	E caballus MN503280.1 Russia	.	.	A	.	.	.	.	T	.	.	.	.	.	.	.	.	.	.	.	T	.	.	.	.	.	.	.	.	.	.	-	.	.	.	.
47	L	E caballus KF038165.1 Mongolia	.	.	A	.	.	.	T	.	.	.	.	.	.	.	.	.	.	.	.	T	T	G	T	.	.	.	.	.	.	.	-	C	.	.	.
48	L	E caballus MW534083.1 Mongolia ancient	.	.	A	.	.	.	T	.	.	.	.	.	.	.	.	.	.	.	.	T	T	G	T	.	.	.	.	.	.	.	-	C	.	.	.
49	L	E caballus MG001445.1 China	.	.	A	.	.	.	T	.	.	.	.	.	.	.	.	.	.	.	.	T	T	G	T	.	.	.	.	.	.	.	-	C	.	.	.
50	L	E caballus MW534082.1 Mongolia ancient	.	.	A	.	.	.	T	.	.	.	.	.	.	.	.	.	.	.	.	T	T	G	T	.	.	.	.	.	.	.	-	C	.	.	.
51	L	E caballus MG001424.1 China	.	.	A	.	.	.	T	.	.	.	.	.	.	.	.	.	.	.	.	T	T	G	T	.	.	.	.	.	.	.	-	C	.	.	.
52	L	E caballus JN398421.1 North America	.	.	A	.	.	.	T	.	.	.	.	.	.	.	.	G	.	.	.	T	T	G	T	.	.	.	.	.	.	.	-	C	.	.	.
53	L	E caballus JN398432.1 Italy	.	.	A	.	.	.	T	.	.	.	.	.	.	.	.	.	.	.	.	T	T	G	T	.	.	.	.	.	.	.	-	C	.	.	.
54	L	E caballus JN398422.1 Central Asia	.	.	A	.	.	.	T	.	.	.	.	.	.	.	.	.	.	.	.	T	T	G	T	.	.	.	.	.	.	.	-	C	.	.	.
55	M	E caballus MG001447.1 China	.	.	A	.	.	.	T	.	.	.	T	.	.	.	.	G	.	.	T	.	.	G	.	.	.	.	.	.	.	.	-	C	.	A	.
56	M	E caballus JN398436.1 Middle East	.	.	A	.	.	.	T	.	.	.	T	.	.	.	.	G	.	.	T	.	.	G	.	.	.	.	.	.	.	.	-	C	.	A	.
57	M	E caballus JN398439.1 Northern Europe	.	.	A	.	.	.	T	.	.	.	T	.	.	.	.	G	.	.	T	.	.	G	.	.	.	.	.	.	.	.	-	C	.	A	.
58	N	E caballus JN398442.1 Northern Europe	.	.	A	.	.	.	T	.	.	.	T	.	.	.	.	.	T	.	T	.	.	G	.	.	C	.	.	.	.	.	-	C	.	.	.
59	N	E caballus KT985980.1 Russia	C	.	A	.	.	.	T	.	.	.	T	.	.	.	.	G	T	.	T	.	.	G	.	.	C	.	.	.	.	.	-	C	.	.	.
60	N	E caballus MG001439.1 China	.	.	A	.	.	.	T	.	.	.	T	.	.	.	.	G	T	.	T	.	.	G	.	.	C	.	.	.	.	.	-	C	.	.	.
61	N	E caballus JN398443.1 Spain	.	.	A	.	.	.	T	.	.	.	T	.	.	.	.	G	T	.	T	.	.	G	.	.	C	.	.	.	.	.	-	C	.	.	.
62	O	E caballus AF014410.2 Korea Jeju	C	.	A	.	.	.	T	.	.	G	.	.	G	.	.	.	.	.	.	.	.	G	.	.	.	.	.	G	.	.	C	.	.	.	.
63	O	E caballus AF014415.2 Mongolia	C	.	A	.	.	.	T	.	.	G	.	.	G	.	.	.	.	.	.	.	.	G	.	.	.	.	.	G	.	.	C	.	.	.	.
64	O	E caballus JN398447.1 Middle East	C	.	A	.	.	.	T	.	.	G	.	.	G	.	.	.	.	.	.	.	.	G	.	.	.	.	.	G	.	.	C	.	.	.	.
65	O	E caballus JN398445.1 Iran	C	.	A	.	.	.	T	.	.	G	.	.	G	.	.	.	.	.	.	.	.	G	.	.	.	.	.	G	.	.	C	.	.	.	.
66	P	E caballus KF038160.1 Korea Jeju	C	.	A	.	.	.	T	.	.	G	.	.	G	.	.	.	.	.	.	.	.	G	.	.	.	.	.	G	.	.	C	.	.	.	.
67	P	E caballus AF056071.1 Mongolia	C	.	A	.	.	.	T	.	.	G	.	.	G	.	.	.	.	.	.	.	.	G	T	.	.	.	.	.	.	.	C	.	.	.	.
68	P	E caballus JN398446.1 Iran	C	.	A	.	.	.	T	.	.	G	.	.	G	.	.	.	.	.	.	.	.	G	.	.	.	.	.	G	.	.	C	.	.	.	.
69	Q	E caballus OM222617.1 Korea Gongpyeongdong ancient	C	.	A	.	G	.	T	.	.	G	.	.	.	T	.	.	.	.	.	.	.	G	.	.	.	.	G	G	.	C	C	.	.	.	.
70	Q	E caballus KF038159.1 Korea Jeju	C	.	A	.	G	.	T	.	.	G	.	.	.	T	.	.	.	.	.	.	.	G	.	.	.	.	G	G	.	C	C	.	.	.	.
71	Q	E caballus JN398455.1 Iran	C	.	A	.	G	.	T	.	.	G	.	.	.	T	.	.	.	.	.	.	.	G	.	.	.	.	G	G	.	C	C	.	.	.	.
72	Q	E caballus MG001421.1 China	C	.	A	.	G	.	T	.	.	G	.	.	.	T	.	.	.	.	.	.	.	G	.	.	.	.	G	G	.	C	C	.	.	.	.
73	Q	E caballus JN398450.1 Central Asia	C	.	A	.	G	.	T	.	.	G	.	.	.	T	.	.	.	.	.	.	.	G	.	.	.	.	G	G	.	C	T	.	.	.	.
74	Q	E caballus MG001418.1 China	C	.	A	.	G	.	T	.	.	G	.	.	.	T	.	.	.	.	.	.	.	G	.	.	.	.	G	G	.	C	C	.	.	.	.
75	Q	E caballus JN398452.1 Central Asia	C	.	A	.	G	.	T	.	.	G	.	.	.	T	.	.	.	.	.	.	.	G	.	.	.	.	G	G	.	C	C	.	.	.	.
76	Q	E caballus MG001419.1 China	C	.	A	.	G	.	T	.	.	G	.	.	.	T	.	.	.	.	.	.	.	G	.	.	.	.	G	G	.	.	C	.	.	.	.
77	Q	E caballus AF014414.2 Mongolia	C	.	A	.	G	.	T	.	.	G	.	.	.	T	.	.	.	.	.	.	.	G	.	.	.	.	G	G	.	.	C	.	.	.	.
78	Q	E caballus AF014406.2 Korea Jeju	C	.	A	.	G	.	T	.	.	G	.	.	.	T	.	.	.	.	.	.	.	.	.	.	.	.	G	G	.	.	C	.	.	.	.
79	R	E caballus JN398456.1 Italy	C	.	A	.	.	T	.	T	C	.	T	.	.	.	.	G	.	.	.	.	.	G	T	C	.	.	.	.	.	.	-	.	.	.	.
80	R	E caballus JN398457.1 Iran	C	.	A	.	.	T	.	T	C	.	T	.	.	.	.	G	.	.	.	.	.	G	T	C	.	.	.	.	.	.	-	.	.	.	.
